# Supplementary material for: Mapping the topographic organization of the human zona incerta using diffusion MRI
Source: eLife. 2026 Oct 23;14:RP103530. doi: 10.7554/eLife.103530 (PMC13694098; doi:10.7554/eLife.103530)
Supplement: Supplementary file 2 [file elife-103530-supp1.docx]

**Supplementary File 1 - Top 5% of connections between each zona incerta (ZI) cluster (k=6) and cortical regions.** Cortical regions from the left and right hemispheres (HCP-MMP1.0) with streamline counts exceeding the 95^th^ percentile for each cluster are ranked in descending order. The source files used for generating these rankings are available in the code repository referenced in the manuscript.

| **Left** |  | **Cluster 1** | | **Cluster 2** | | **Cluster 3** | | **Cluster 4** | | **Cluster 5** | | **Cluster 6** | |
| --- | --- | --- | --- | --- | --- | --- | --- | --- | --- | --- | --- | --- | --- |
|  |  | *HCP-MMP* | *Brodmann* | *HCP-MMP* | *Brodmann* | *HCP-MMP* | *Brodmann* | *HCP-MMP* | *Brodmann* | *HCP-MMP* | *Brodmann* | *HCP-MMP* | *Brodmann* |
|  | **1** | PeEc | Temporal | 10d | BA10 | 8BL | BA08 | 6ma | BA06 | 4 | BA04 | 1 | BA01 |
|  | **2** | TGd | Temporal | a10p | BA10 | SFL | BA06 | SFL | BA06 | 6mp | BA06 | 3b | BA03 |
|  | **3** | 10d | BA10 | a47r | BA47 | 9m | BA09 | 6mp | BA06 | 3b | BA03 | 4 | BA04 |
|  | **4** | Pir | Temporal | 10pp | BA10 | 8BM | BA08 | 4 | BA04 | 1 | BA01 | 2 | BA02 |
|  | **5** | a10p | BA10 | 9m | BA09 | 10d | BA10 | SCEF | BA06 | 3a | BA03 | 3a | BA03 |
|  | **6** | V1 | V1 | p10p | BA10 | 9a | BA09 | 6d | BA06 | 6d | BA06 | 7AL | BA07 |
|  | **7** | PreS | Temporal | 9a | BA09 | a47r | BA47 | 8BL | BA08 | 6ma | BA06 | 7Am | BA07 |
|  | **8** | a47r | BA47 | 11l | BA11 | 6ma | BA06 | 6a | BA06 | 5m | BA06 | 5L | BA05 |
|  | **9** | 10pp | BA10 | 8BL | BA08 | 9p | BA09 | 8BM | BA08 | 2 | BA02 | 5m | BA05 |
|  |  |  |  |  |  |  |  |  |  |  |  |  |  |
| **Right** |  | **Cluster 1** | | **Cluster 2** | | **Cluster 3** | | **Cluster 4** | | **Cluster 5** | | **Cluster 6** | |
|  |  | *HCP-MMP* | *Brodmann* | *HCP-MMP* | *Brodmann* | *HCP-MMP* | *Brodmann* | *HCP-MMP* | *Brodmann* | *HCP-MMP* | *Brodmann* | *HCP-MMP* | *Brodmann* |
|  | **1** | 10pp | BA10 | 10pp | BA10 | 8BL | BA08 | 6ma | BA06 | 4 | BA04 | 4 | BA04 |
|  | **2** | a10p | BA10 | a10p | BA10 | SFL | BA06 | SFL | BA06 | 6mp | BA06 | 3b | BA03 |
|  | **3** | V1 | V1 | 10d | BA10 | 9m | BA09 | 6mp | BA06 | 3b | BA03 | 1 | BA01 |
|  | **4** | TGd | Temporal | 9a | BA09 | 9a | BA09 | 4 | BA04 | 3a | BA03 | 3a | BA03 |
|  | **5** | PeEc | Temporal | 9m | BA09 | 8BM | BA08 | SCEF | BA06 | 1 | BA01 | 2 | BA02 |
|  | **6** | 10d | BA10 | a47r | BA47 | 6ma | BA06 | 8BL | BA08 | 6ma | BA06 | 5L | BA05 |
|  | **7** | 8BL | BA08 | p10p | BA10 | 10d | BA10 | 6d | BA06 | 6d | BA06 | 7AL | BA07 |
|  | **8** | 9m | BA09 | 8BL | BA08 | a47r | BA47 | 8BM | BA08 | SFL | BA06 | 5m | BA05 |
|  | **9** | 9a | BA09 | 11l | BA11 | a10p | BA10 | s6-8 | BA06/BA08 | SCEF | BA06 | 7Am | BA07 |
