# Supplementary material for: Mapping the topographic organization of the human zona incerta using diffusion MRI
Source: eLife. 2026 Oct 23;14:RP103530. doi: 10.7554/eLife.103530 (PMC13694098; doi:10.7554/eLife.103530)
Supplement: Peer review history

# Editor report: eLife Assessment

**DOI:** 10.7554/eLife.103530.3.sa0

**Authors:** Alex Fornito

**Affiliations:** Monash University Australia

**Keywords:** Compelling, Important

This **important** study uses diffusion magnetic resonance imaging to non-invasively map the white matter fibres connecting the zona incerta and cortex in humans. The authors present **compelling** evidence to indicate that these connections are organized along a rostro-caudal axis. The findings will be of interest to researchers interested in neuroanatomy and cortico-subcortical connectivity.

# Referee report: Reviewer #1 (Public review)

**DOI:** 10.7554/eLife.103530.3.sa1

Summary:

This is a study which used 7T diffusion MRI in subjects from a Human Connectome Project dataset to characterize the zona incerta, an area of gray matter whose involvement has been demonstrated in a broad range of behavioral and physiologic functions. The authors employ tractography to model white matter tracts that involve connections with the ZI and use clustering techniques to segment the ZI into distinct subregions based on similar patterns of connectivity. The authors report a rostral-caudal organization of the ZI's streamlines where rostrally-projecting tracts are rostrally-positioned in the ZI and caudally-projecting tracts are caudally-positioned in the ZI.

Strengths:

The paper presents robust findings that demonstrate subregions of the human ZI that appear to be structurally distinct using a combination of spectral clustering and diffusion map embedding methods. The results of this work can contribute to our understanding of the anatomy and structural connectivity of the ZI, allowing us to further explore its role as a neuromodulatory target for various neurological disorders.

Weaknesses:

There should be further discussion of the clustering methods employed and why they are appropriate for the pertinent data. Additionally, the limitations of analyzing solely the cortical connections of the zona incerta should be addressed, as anatomical studies of the ZI have shown significant involvement of the ZI in tracts projecting to deep brain regions.

Comments on the latest version:

I reviewed the file and am more than satisfied with the authors responses and edits.

# Referee report: Reviewer #2 (Public review)

**DOI:** 10.7554/eLife.103530.3.sa2

Summary:

Haast et al. investigated the organization of the zona incerta (ZI) in the human brain based on its structural connectivity to the neocortex. They found that the ZI is organized according to a primary rostro-caudal gradient, where the rostral ZI is more strongly connected to the prefrontal cortex and the caudal ZI to sensorimotor cortex. They also found that the central region of the ZI is differently connected to neocortex compared with the rostral and caudal regions and could be important as a deep brain stimulation target for the treatment of essential tremor.

Strengths:

I think the overall quality of this work is great, and the results are presented in a very clear and organized manner. I particularly appreciate the effort that the authors put into validating the results using 7T and 3T data, as well as test-retest data.

Weaknesses:

The initial version of the manuscript left me with a couple of minor concerns that the authors addressed in the revised version. These were related to the clinical relevance of the work (now addressed with the lower-resolution data), and to the initial emphasis on a dorso-ventral gradient that the data did not strongly support.

# Author comment: Author response

**DOI:** 10.7554/eLife.103530.3.sa3

**Authors:** Roy AM Haast, Jason Kai, Alaa Taha, Violet Liu, Loxlan W Kasa, Greydon Gilmore, Maxime Guye, Ali R Khan, Jonathan C Lau

**Affiliations:** Aix-Marseille Université Marseille France; Child Mind Institute London Canada; Western University London Canada; Western University London Canada; Western University London Canada; Western University London Canada; Aix-Marseille Université Marseille France; Western University London Canada; Western University London Canada

The following is the authors’ response to the original reviews.

**Public Reviews:**

**Reviewer #1 (Public review):**

Summary:

This is a study that used 7T diffusion MRI in subjects from a Human Connectome Project dataset to characterize the zona incerta, an area of gray matter whose involvement has been demonstrated in a broad range of behavioral and physiologic functions. The authors employ tractography to model white matter tracts that involve connections with the ZI and use clustering techniques to segment the ZI into distinct subregions based on similar patterns of connectivity. The authors report a rostral-caudal organization of the ZI's streamlines where rostrally-projecting tracts are rostrally-positioned in the ZI and caudally-projecting tracts are caudally-positioned in the ZI.

Strengths:

The paper presents robust findings that demonstrate subregions of the human ZI that appear to be structurally distinct using a combination of spectral clustering and diffusion map embedding methods. The results of this work can contribute to our understanding of the anatomy and structural connectivity of the ZI, allowing us to further explore its role as a neuromodulatory target for various neurological disorders.

Weaknesses:

There should be further discussion of the clustering methods employed and why they are appropriate for the pertinent data. Additionally, the limitations of analyzing solely the cortical connections of the zona incerta should be addressed, as anatomical studies of the ZI have shown significant involvement of the ZI in tracts projecting to deep brain regions.

We are grateful to the reviewer for recognizing the strengths of our study, as well as for providing constructive suggestions to further strengthen the manuscript.

In response to the reviewer’s feedback, we have expanded our discussion of the clustering methods employed, including the rationale for using spectral clustering in combination with diffusion map embedding, and clarified why this approach is well-suited to connectivity-based parcellation of the ZI.

Additionally, we have expanded the Discussion to address the limitations of focusing exclusively on cortical connections. As the reviewer correctly notes, anatomical studies have demonstrated that the ZI has extensive connections with deep brain regions, and our approach therefore represents only a partial view of its connectivity. We have previously demonstrated the feasibility of reconstructing subcortical pathways using in vivo diffusion MRI (Kai et al., NeuroImage, 2022), providing a foundation for extending the present framework beyond cortical connectivity. However, as iterated below in our specific response to reviewer 1, we believe this deserves a separate thorough investigation. Nonetheless, we now explicitly discuss this limitation in the revised Discussion and outline directions for future work incorporating subcortical connectivity analyses.

**Reviewer #2 (Public review):**

Summary:

Haast et al. investigated the organization of the zona incerta (ZI) in the human brain based on its structural connectivity to the neocortex. They found that the ZI is organized according to a primary rostro-caudal gradient, where the rostral ZI is more strongly connected to the prefrontal cortex and the caudal ZI to the sensorimotor cortex. They also found that the central region of the ZI is differently connected to the neocortex compared with the rostral and caudal regions, and could be important as a deep brain stimulation target for the treatment of essential tremors.

Strengths:

I think the overall quality of this work is great, and the results are presented in a very clear and organized manner. I particularly appreciate the effort that the authors put into validating the results using 7T and 3T data, as well as test-retest data.

Weaknesses:

That being said, I was left with a couple of concerns after reading the paper.

- Although the authors discussed animal evidence for a dorsal-ventral organization of the ZI, I thought that the evidence they presented for it in this paper was not so convincing. In Figure S5, the second gradient (G2) shows a clear dorsoventral pattern, but this pattern seems to primarily separate the ZI and H fields rather than show an internal topology of the ZI. This is more likely the case given that there are two bands (superior and inferior) of high G2 values surrounding a single band (middle) of low G2 values. The evidence for the rostrocaudal gradient, on the other hand, is quite convincing.

- HCP data is still too advanced for clinical translation. Although 3T is becoming more and more prevalent for presurgical planning, the HCP 3T dataset is acquired with a voxel size of 1.25mm, which is a far higher resolution than the typical clinical scan. It would be very useful for clinical readers to see what individual subject replicability looks like if the data were acquired at the more typical voxel size of 2mm. This could be achieved by replicating the analysis on a downsampled version of the HCP data that more closely resembles clinical data. This is understandably a large undertaking, so it could be left to future validation work.

We thank the reviewer for their positive evaluation of our work and for highlighting the clarity of the results, and our validation efforts across 7T, 3T, and test-retest datasets.

Regarding the reviewer’s concern about the evidence for a dorsal-ventral organization, we agree that the rostro-caudal gradient is more prominent and convincing in our data, while the dorsal-ventral pattern is less robust. As the reviewer points out, the second gradient (G2) in Figure S5 may primarily reflect differences between the ZI and surrounding H fields, rather than a clear internal subdivision within the ZI itself. We have revised the Discussion to clarify this interpretation, emphasizing that our evidence for a dorsal-ventral organization is more tentative and requires further validation, particularly in light of prior animal literature.

We also appreciate the reviewer’s important point regarding clinical translation. Indeed, the HCP datasets, both at 7T and 3T, use acquisition parameters (e.g., 1.25 mm voxel size at 3T) that exceed those of typical clinical scans. We therefore assessed the replicability of our findings in data acquired at more clinically representative resolutions (i.e., 2 mm voxel size at 3T). Details concerning this analysis are outlined in our response to reviewer 2 below.

**Recommendations for the authors:**

**Reviewer #1 (Recommendations for the authors):**

(1) When using "spectral clustering" to segment the ZI per its structural connectivity, it is unclear how k=6 clusters were chosen. Moreover based on previous reports of rodent ZI cytoarchitecture into rostral, dorsal, ventral, and caudal regions there is disagreement with the topographic organization of six clusters presented in this manuscript. Moreover, "diffusion map embedding" was not described or cited. This technique of dimensionality reduction and how it was applied to the data should be specifically described.

We thank the reviewer for this thoughtful comment. We agree that selecting the optimal number of clusters in data-driven approaches such as spectral clustering is inherently challenging in the absence of a definitive ground truth. To address this, we computed alternative cluster solutions across a range of k values (k=2-8), which are presented in Supplementary Figure 2B. Our decision to focus on k=6 was guided by prior cytoarchitectonic descriptions of the rodent ZI by Romanowski et al., 1985, who delineated six distinct sectors (pars rostropolaris, pars dorsalis, pars ventralis, pars magnocellularis, pars retropolaris, and pars caudalis). Thus, our approach aligns the data-driven clustering with established anatomical subdivisions. Additionally, we found that k=6 provided a meaningful level of granularity for probing location-dependent neuromodulatory effects within the ZI, as discussed in the revised Discussion section (‘Discrete subregions of the zona incerta using spectral clustering’, second paragraph).

Finally, we acknowledge the lack of clarity concerning “diffusion map embedding” in the original submission. We have now more explicitly mentioned diffusion map embedding in the “Connectivity gradients” paragraph in the Methods section, which includes the relevant references as well as description on how it was applied to the data.

(2) "Strong correlations with cognitive terms and cortical hierarchies were particularly evident for clusters 3-5 (Figure 7a-b), which have the highest number of connecting streamlines (Figure 3c). Cluster 5, located near the central sulcus, is significantly linked to movement related cognitive processing (Pearson's r = 0.623, p < 0.005) and CogPC1 (Pearson's r = 0.593, p < 0.005). Cluster 4, situated more anteriorly, overlaps with regions involved in working memory (Pearson's r = 0.494, p < 0.001) with a high level of expression of serotonin (5-HT1B) receptors (Pearson's r = 0.413, p < 0.005) (Beliveau et al., 2017). Cluster 3, located further towards the frontal pole, is associated with mood (Pearson's r = 0.473, p < 0.005) and impulsivity (Pearson's r = 0.473, p < 0.001), and the sensorimotor association axis (Pearson's r = 0.583, p < 0.005) (Sydnor et al., 2021). Clusters 1, 2, and 6, characterized by the least number of connecting streamlines (Figure 3c), were relatively weakly associated (i.e., low Spearman's coefficient and/or within the spatial autocorrelation range) with cognitive terms or cortical hierarchies."

The validity of identifying correlations with the spectral clusters and functional connectivity in tasks related to "keywords", and reporting similarities between these data and the regions most strongly connected via tractography, is a bit questionable. These results are based on studies that may not report all relevant findings. There is a bias towards regions that are more commonly studied with fMRI. Moreover, claims can be made about assigning functions specific to a brain region for almost any structure/function relationship (with some exceptions).

We agree that correlations between connectivity-defined ZI clusters and functional annotations derived from external datasets should be interpreted with caution, given potential biases in the available literature (e.g., overrepresentation of well-studied cortical regions in fMRI meta-analyses) and the inherent risk of over-assigning functions to structural subdivisions. Our intention was not to make definitive claims about the functional specialization of individual ZI subregions, but rather to provide an exploratory framework for situating the ZI within broader cortical hierarchies and functional domains. These analyses are intended to generate hypotheses and to offer preliminary insight into how connectivity-based subdivisions of the ZI may relate to cognition and behavior.

We agree with the reviewer that future studies should be specifically designed to address these questions more directly, for example, by combining connectivity-informed parcellations of the ZI with task-based or resting-state fMRI in the same subjects. Such targeted approaches will be necessary to rigorously establish the integration of the ZI within the brain’s functional organization.

(3) The ZI's connections to many subcortical structures have also been reported in rodents and non-human primates. Moreover, the authors describe the efficacy of DBS of the caudal ZI in alleviating symptoms in patients with essential tremor, which indicates modulation of the dentato-rubro-thalamic tract fibers that project to subcortical structures such as the VIM thalamus, red nucleus, and cerebellum. The atlas in the study was characterized per the ZI's cortical connections only. These concerns should be addressed in the discussion.

We agree with the reviewer that incorporating subcortical connections is essential for a comprehensive understanding of ZI connectivity. Building on our prior work demonstrating the feasibility of subcortical tractography (Kai et al., NeuroImage, 2022), we propose a systematic investigation of in vivo subcortico-incertal tractography as a critical next step. We believe that a dedicated investigation is required to systematically evaluate subcorticoincertal tractography, optimize reconstruction of key pathways (e.g., the dentato-rubrothalamic tract), and determine how these subcortical connections contribute to the topographic organization of the human ZI. We now explicitly discuss these considerations and identify them as an important direction for future work. Such (currently ongoing) work will not only clarify how subcortical inputs shape the topography of the ZI, but will also enable targeted optimization of tractography parameters to maximize the reliable reconstruction of specific but key pathways, including the dentato-rubro-thalamic tract. We believe this line of investigation will be important in advancing both the anatomical characterization and translational relevance of the ZI.

**Reviewer #2 (Recommendations for the authors):**

(1) Re: data quality compared to the clinic, this could be achieved by replicating the analysis on a downsampled version of the HCP data that more closely resembles clinical data. This is understandably a large undertaking, so it could be left to future validation work.

We thank the reviewer for this valuable suggestion. While this was suggested as potential future work, we felt adding this analysis would strengthen the manuscript. In response, we repeated our analyses using diffusion MRI data that more closely approximates a clinical acquisition with a lower spatial resolution (i.e., 2 mm vs. 1.25 mm isotropic) and number of diffusion-encoding directions (i.e., 130 vs. 270, Kasa et al., NeuroImage Clin., 2022). We have included these analyses in the revised manuscript.

Reassuringly, the principal rostro-caudal gradient of cortico-incertal connectivity was preserved, demonstrating that the dominant organizational feature of the ZI is robust even under clinically representative acquisition conditions. However, finer-grained parcellations were less consistent with the original HCP analyses. In particular, cluster solutions with larger numbers of clusters (k > 3) became increasingly variable, indicating that differentiation of subtle connectivity-defined subregions benefits from the higher spatial and angular resolution afforded by research-grade diffusion MRI.

We believe these findings provide a more nuanced assessment of the translational potential of our approach. They suggest that the large-scale topographic organization of the ZI can be recovered using clinically realistic diffusion MRI, while also highlighting the current limitations of routine clinical acquisitions for resolving finer anatomical subdivisions.

We have incorporated these results and their implications into the revised manuscript.

(2) Figure 6 legend labels: (c) and (d) should be (b) and (c).

Thank you for highlighting this discrepancy. We have corrected as proposed.
